# Individual Differences in Automatic Emotion Regulation Interact with Primed Emotion Regulation during an Anger Provocation

**DOI:** 10.3389/fpsyg.2017.00614

**Published:** 2017-04-21

**Authors:** Jing Zhang, Ottmar V. Lipp, Ping Hu

**Affiliations:** ^1^Department of Psychology, Renmin University of ChinaBeijing, China; ^2^School of Psychology and Speech Pathology, Curtin UniversityPerth, WA, Australia

**Keywords:** automatic emotion regulation, priming, skin conductance, heart rate, anger

## Abstract

The current study investigated the interactive effects of individual differences in automatic emotion regulation (AER) and primed emotion regulation strategy on skin conductance level (SCL) and heart rate during provoked anger. The study was a 2 × 2 [AER tendency (expression vs. control) × priming (expression vs. control)] between subject design. Participants were assigned to two groups according to their performance on an emotion regulation-IAT (differentiating automatic emotion control tendency and automatic emotion expression tendency). Then participants of the two groups were randomly assigned to two emotion regulation priming conditions (emotion control priming or emotion expression priming). Anger was provoked by blaming participants for slow performance during a subsequent backward subtraction task. In anger provocation, SCL of individuals with automatic emotion control tendencies in the control priming condition was lower than of those with automatic emotion control tendencies in the expression priming condition. However, SCL of individuals with automatic emotion expression tendencies did no differ in the automatic emotion control priming or the automatic emotion expression priming condition. Heart rate during anger provocation was higher in individuals with automatic emotion expression tendencies than in individuals with automatic emotion control tendencies regardless of priming condition. This pattern indicates an interactive effect of individual differences in AER and emotion regulation priming on SCL, which is an index of emotional arousal. Heart rate was only sensitive to the individual differences in AER, and did not reflect this interaction. This finding has implications for clinical studies of the use of emotion regulation strategy training suggesting that different practices are optimal for individuals who differ in AER tendencies.

## Introduction

Automatic emotion regulation (AER) is characterized by goal-driven changes to one's emotional experience without making a conscious decision to do so and without engaging in deliberate control (Mauss et al., 2007a). The positive effects of AER have been documented across a number of studies. For example, when anger was provoked by the experimenter who asked participants to perform a tedious counting task, individuals with an automatic tendency to control emotion differed from those with a tendency to express emotion. Participants who controlled emotion experienced less anger, fewer negative thoughts and greater sympathetic activation, greater cardiac output, no effects on mean arterial blood pressure, and lower total peripheral resistance, compared with automatic emotion expression participants (Mauss et al., 2006, 2007b). Christou-Champi et al. (2015) found that structured practice of emotional reappraisal spontaneously decreased the time required to regulate emotions and increased heart rate variability, a positive change that was maintained even 2 weeks after the practice had been completed. The auto-motive model provides a potential explanation of how AER works. When goals or norms referring to regulating emotions are formed in an individual's mind, they can be activated without his or her awareness and influence an emotional response (Fitzimons and Bargh, 2004; Koole and Coenen, 2007; Mauss and Tamir, 2014; Kobylińska and Karwowska, 2015).

Evidence that automatic emotion control, as indicated by the performance on an emotion regulation-IAT (ER-IAT), has positive effects on emotion regulation has been found in studies involving measures as diverse as resting prefrontal spontaneous alpha asymmetry and self-reported (Jackson et al., 2003; Mauss et al., 2006; Drabant et al., 2009). Moreover, this pattern of results has emerged in correlation analyses of self-reported emotional experience, behavior, peripheral physiology, and performance on the ER-IAT, and in factorial designs that compared the performance of groups defined as favoring automatic emotion control tendencies or automatic emotion expression tendencies based on their scores on the control and expression dimensions in the ER-IAT (Mauss et al., 2006). These studies provided evidence that automatic emotion control decreases feelings of anger and is associated with an adaptive pattern of cardiovascular responding. Thus, this line of research has so far suggested that individual differences in the tendency to control emotions influence the experience of and physiological response to situations that are likely to elicit a particular emotional response.

An increasing number of studies have also examined AER which is manipulated by priming technology. Different emotion regulation strategies can be primed using a sentence unscrambling task or the aversive emotional states provocation (Mauss et al., 2007b; Williams et al., 2009; DeWall et al., 2011; Vogt et al., 2011). Williams et al. (2009), for instance, primed participants with either a goal to control emotion using reappraisal or neutral concepts, and found that, participants primed with reappraisal goals exhibited a larger decrease in heart rate during a subsequent anxiety-eliciting task. Mauss et al. (2007b) used a sentence unscrambling task to prime different emotion regulation concepts and found that participants primed to control emotion reported less anger and displayed more adaptive cardiovascular responding than did participants primed to express emotion.

However, little is known about whether individual differences in AER and emotion regulation strategies that are induced experimentally interact. Clarifying the interaction of individual difference in AER and emotion regulation priming, will help to explain some of the apparently inconsistent findings in the literature. For example, Williams et al. (2009) found that effects of reappraisal priming seemed most pronounced for those who did not use reappraisal strategies habitually. This finding may suggest an interaction between AER priming and individual differences in AER. Furthermore, an interaction between individual differences in AER and emotion regulation priming, has implications for clinical studies using emotion regulation strategy training. These interventions could be made more efficient by assigning participants to different training conditions.

In response to an anger provocation, heart rate, which is an index of joint sympathetic and parasympathetic activation, should increase relative to baseline (Campbellsills et al., 2006; Hofmann et al., 2009; Herrero et al., 2010). Skin conductance level (SCL) is a reliable index of emotional arousal and arousal changes (Boucsein, 1992; Lang, 1995; Khalfa et al., 2002; Zhang et al., 2011). When manipulating AER by priming regulation goals or when measuring individual difference in AER, self-reports of anger decreased when automatic emotion control occurred (Mauss et al., 2006, 2007b).

Thus, the present study was designed to examine whether individual differences in AER interact with the effects of experimental AER priming during an anger provocation. In the present study, participants were differentiated into those with an automatic emotion control tendency and those with an automatic emotion expression tendency based on their performance on an emotion regulation-IAT. The ER-IAT measures an implicit positive evaluation or attitude toward one tendency of emotion regulation (Mauss et al., 2006). Positive concepts appear to enhance individuals' pursuit of these goals (Custers and Aarts, 2005). A relatively stronger association between emotion control and positive items in the ER-IAT might predicate a greater likelihood of engaging in automatic emotion control. The utility of the ER-IAT in the study of AER has been demonstrated by many findings, such that positive implicit evaluation of emotion control tendencies is associated with automatic, successful, and physiologically adaptive emotion regulation (Mauss et al., 2006), greater level of psychological health (Hopp et al., 2011) and no hemispheric asymmetry of the LPP component at posterior electrodes (a right dominance of the LPP component at posterior electrodes, Zhang and Zhou, 2014).

In the present study, half the participants in each group were exposed to an emotion control priming condition and whereas the others were exposed to an emotion expression priming condition. All participants were subjected to a subsequent anger provocation by being blamed for slow performance during a backward subtraction task. Based on previous findings of studies examining individual differences in emotion regulation, we predicted that individual differences in AER will modulate the effects of emotion regulation priming. It is hypothesized that, in response to an anger provocation, participants with an automatic emotion control tendency in the control priming condition (AECT/CP) will show the lowest autonomic activation (lowest SCL and heart rate) and participants with an automatic emotion expression tendency in the expression priming condition (AEET/EP) will show the highest autonomic activation. Participants with an automatic emotion control tendency in the expression priming condition (AECT/EP) and participants with an automatic emotion expression tendency in the control priming condition (AEET/CP) are expected to show intermediate levels of autonomic activation.

## Methods

### Ethics statement

The protocol of this study was approved by the Institutional Review Board of the Department of Psychology of Renmin University of China. Informed written consent was obtained from each subject before the experiment.

### Participants

Ninety-four students (*M* = 21, *SD* = 4.4, 46 males, and 48 females) from Renmin University of China volunteered participation in the study and were compensated with 30 Chinese Yuan. Forty-eight participants were selected into the automatic emotion control group and 48 participants were assigned to the automatic emotion expression group (Criterion: emotion control score on the Emotion Regulation-Implicit Association Test [ER-IAT] larger or smaller than zero, respectively). Of the 48 participants with automatic emotion control tendencies, half were assigned randomly to receive emotion control priming and half were assigned to receive emotion expression priming. Of the 48 participants with automatic emotion expression tendencies, 24 were randomly assigned to emotion control priming, and 24 to emotion expression priming, however, two participants in the latter group did not complete the experiment leaving 22 in this condition. The 24 participants with automatic emotion control tendency in the emotion control priming condition, included 13 males and 11 females (*M* = 21.2, *SD* = 2.20, range 19–27); the 24 participants with automatic emotion expression tendency in the emotion control priming condition, included 12 males and 12 females (*M* = 21.6, *SD* = 2.43, range 19–27); the 24 participants with automatic emotion control tendency in the emotion expression priming condition, included 12 males and 12 females (*M* = 21.8, *SD* = 1.69, range 19–26); the 22 participants with automatic emotion expression tendency in the emotion expression priming condition, included 11 males and 11 females (*M* = 21.9, *SD* = 2.15, range 19–28). Thus, the experimental design involved four groups, AEET/EP, automatic emotion expression tendency/expression priming; AEET/CP, automatic emotion expression tendency/control priming; AECT/EP, automatic emotion control tendency/expression priming; AECT/CP, automatic emotion control tendency/control priming.

The purpose of the experiment was presented as the assessment of physiological responses during a backward subtraction task. The true aim was explained after the participants finished the experiment. All participants had normal or corrected-to-normal vision, reported no history of mental illness, head injury, or heart disease, and were not currently taking any medication.

### Design

The study involved a 2 × 2 [AER tendency (expression vs. control) × priming (expression vs. control)] between subject factorial design.

### Procedure

Participants arrived at the laboratory individually and completed the Chinese version of the Emotion Regulation- Implicit Association Test (ER-IAT), which was adapted from Mauss et al. (2006). Participants were told that they should categorize each stimulus word as quickly as possible and with no errors. The ER-IAT contained five blocks. In Block 1, the participants practiced categorizing 20 Chinese words as positive or negative. In Block 2, the participants practiced categorizing 20 words into emotion regulation and positive categories or emotion expression and negative categories. In the critical Block 3, the participants repeated this categorization with 40 words. In Block 4, the participants practiced categorizing 20 Chinese words into emotion regulation and negative categories vs. emotion expression and positive categories. In Block 5, the participants repeated this categorization with 40 Chinese words. The words used here were all presented in Appendix B. Participants whose score in the ER-IAT was more (less) than zero were assigned into the automatic emotion control/expression groups respectively. After the ER-IAT, participants watched a neutral film for 3 min as a baseline condition and completed an adapted Chinese version of the PANAS that included an item referring to anger (Watson et al., 1988; Qiu et al., 2009).

A sentence unscrambling task was used to prime emotion regulation goals (control or expression). Each participant received 10 sets of five words and was asked to form a sentence using four of the five words provided per set. In the emotion expression priming condition, nine sets contained a word related to emotion expression whereas in the emotion control priming condition nine sets contained a word related to emotion control. All participants also received one filler set.

The 18 emotion control or expression related words used in the priming task were selected from 40 emotion regulation related words rated by 30 undergraduate students who majored in psychology. Nine emotion control-related words (See Appendix A) were selected that received a rating of 3 or more on a 5-point scale for the dimension of emotion control. Another 9 expression-related words (See Appendix A), which received a rating of 3 or more on a 5 points scale for the dimension of emotion expression, were selected as the emotion expression-related words.

### Anger provocation

After the sentence unscrambling task, participants were instructed to complete a backward subtraction task, which required participants to complete three successive subtractions for 1 min each, 7 from 18,652, 7 from 87,611, and 7 from 20,134. The experimenter interrupted participants and blamed them for slow performance while participants were performing the subtraction. The experimenter said “Be quick! You are too slow!!!” This anger provocation task lasted 3 min for each participant. Participants completed the Chinese version of the PANAS (Watson et al., 1988) before and after the subtraction task.

A manipulation check was completed to ensure that participants felt anger in the subtraction task and did not realize the real aim of this experiment. The experimenter asked the participants to complete the Chinese version of the PANAS and to state what they believed the aim of the experiment to be. After the manipulation check, the experimenter explained the true aim of the experiment to the participants.

### Recording

All physiological indices were recorded at a sampling rate of 1,000 Hz with Biopac equipment (Biopac Systems, Inc., Santa Barbara, CA, USA) using Acknowledge 3.9.1 data acquisition and processing software on an IBM-compatible Pentium computer. Skin conduction level and an electrocardiogram (ECG) were recorded continuously from the beginning of the baseline film to the end of the backward subtraction. The ECG was collected using three disposable electrodes, placed on the participant's right forearm, and 10 cm above the right and left ankle. Electrodes were placed after the skin was wiped with alcohol and scrubs. The ECG was recorded with an ECG100C amplifier with a band pass from 0.5 to 35 Hz, and a gain of 500. The ECG was converted off-line to heart rate (HR) in beats per minute by Biopac processing software (Acqknowledge 3.9.1). Skin conductance was recorded with a GSR100C module, amplified to 5 μSiemens/V, with a DC high pass and a 1 Hz low pass filter. Two electrodes filled with electrode gel (GEL100) were applied to the tips of the index and middle fingers after cleaning the skin. To assess the effect of the anger provocation, the mean SCL and heart rate were derived from an interval that started at the beginning of the subtraction and ended after the first blame was delivered.

### Analysis

Separate 2 × 2 factorial ANCOVAs were conducted on anger ratings derived from an adapted Chinese version of the PANAS (PANAS: Watson et al., 1988; Qiu et al., 2009), SCL, and heart rate for data collected after the anger provocation and after priming, using baseline responding as the covariate.

## Results

### Anger ratings

**Figure 1:**
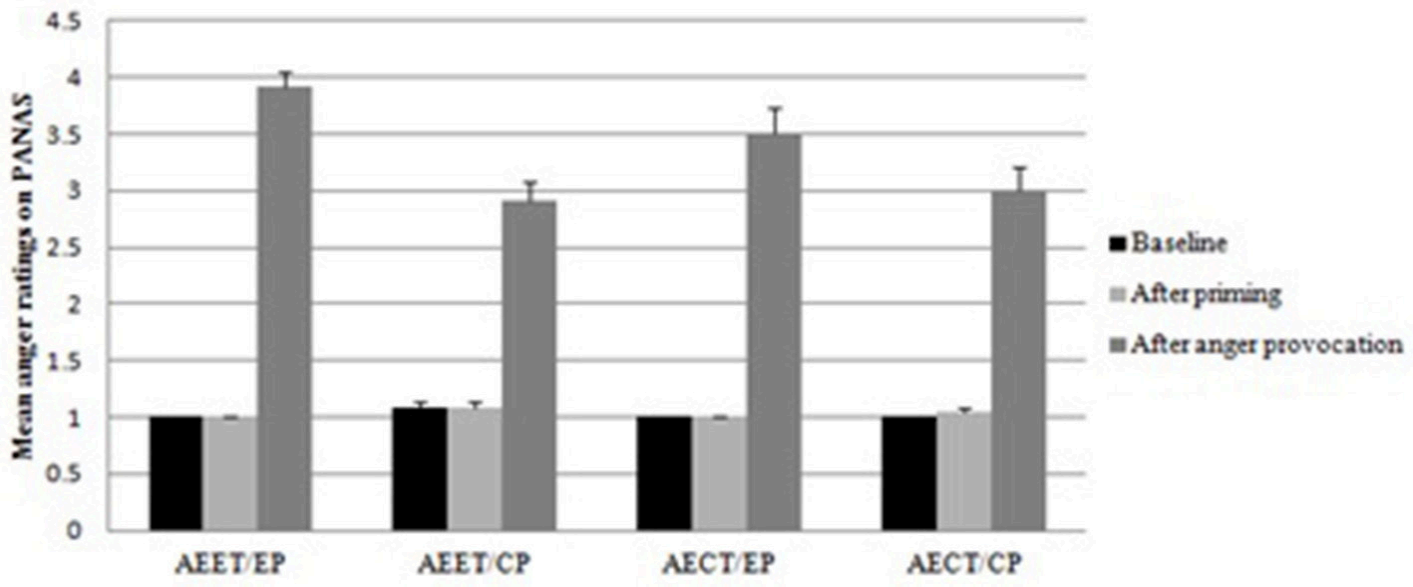
**Mean anger ratings in the adapted vision of the PANAS for the four groups (AEET/EP, automatic emotion expression tendency/expression priming; AEET/CP, automatic emotion expression tendency/control priming; AECT/EP, automatic emotion control tendency/expression priming; AECT/CP, automatic emotion control tendency/control priming) across the three measurement points (baseline, after priming, after anger provocation; error bars represent standard errors of the mean)**.

As shown in Figure 1, a main effect of priming was significant for the PANAS anger ratings collected after the anger provocation, *F*_(1, 94)_ = 14.803, *p* < 0.01, η_*p*_^2^ = 0.143, with higher anger reported in the expression priming condition than in the control priming condition. No other significant effects were found (*ps* < 0.05). The ANCOVA showed no significant effects for the rating data after priming (*ps* < 0.05). The means and SD of anger ratings were shown in Table 1.

**Table 1:** **Means and standard deviations for all the dependent variables after the anger provocation**.

**Group**	**Anger ratings on PANAS Mean (*SD*)**	**Skin conductance level Mean (*SD*)**	**Heart rate Mean (*SD*)**
AEET/EP	3.91 (0.68)	17.33 (9.00)	96.41 (19.73)
AEET/CP	2.92 (0.78)	14.66 (9.80)	95.56 (12.19)
AECT/EP	3.50 (1.14)	13.47 (6.64)	80.87 (11.67)
AECT/CP	3.00 (1.02)	9.53 (6.40)	88.37 (15.90)

### Skin conductance level

**Figure 2:**
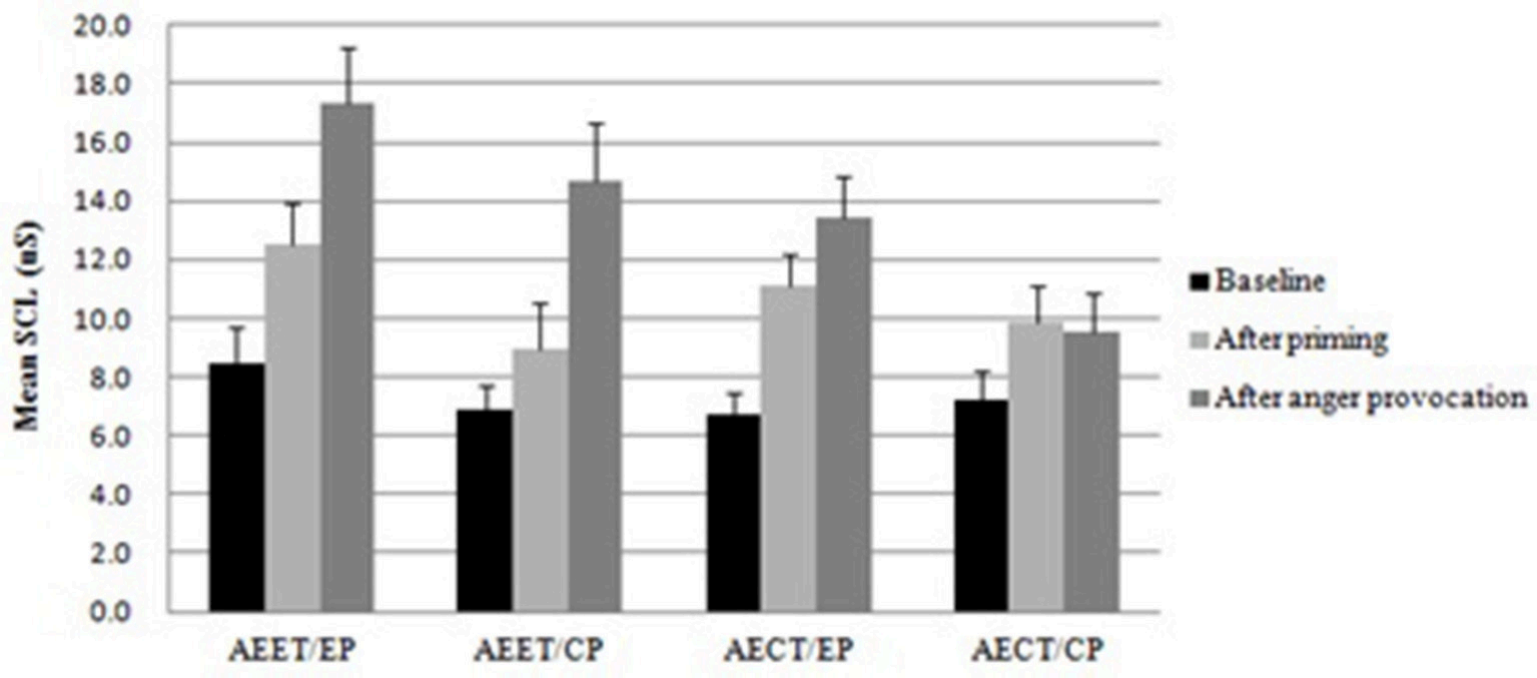
**Mean skin conductance levels for the four groups (AEET/EP, automatic emotion expression tendency/expression priming; AEET/CP, automatic emotion expression tendency/control priming; AECT/EP, automatic emotion control tendency/expression priming; AECT/CP, automatic emotion control tendency/control priming) across the three measurement points (baseline, after priming, after anger provocation; error bars represent standard errors of the mean)**.

An analysis of the SCL data after the anger provocation revealed main effects of priming condition, *F*_(1, 94)_ = 6.192, *p* < 0.05, η_*p*_^2^ = 0.065, with larger SCL in the expression priming condition than in the control priming condition, and AER tendency, *F*_(1, 94)_ = 11.864, *p* < 0.01, η_*p*_^2^ = 0.118, with lager SCL for individuals with automatic emotion expression tendency than those with automatic emotion control tendency. These main effects were qualified by a two way interaction, *F*_(1, 94)_ = 4.428, *p* < 0.05, η_*p*_^2^ = 0.047 (see Figure 2). The simple effect analysis revealed that the SCL was lower in individuals with automatic emotion control tendencies in the control priming condition than in individuals with automatic emotion control tendencies in the expression priming condition (*p* < 0.05). However, the SCL of individuals with automatic emotion expression tendencies showed no difference in the automatic emotion control priming or the automatic emotion expression priming condition (*p* < 0.05).

An analysis of the SCL data after the priming revealed a main effect of priming condition, *F*_(1, 94)_ = 7.983, *p* < 0.01, η_*p*_^2^ = 0.082. The SCL in the expression priming condition was higher than in the control priming condition. No other effects were found (*ps* < 0.05). The means and SD were presented in Table 1.

### Heart rate

**Figure 3:**
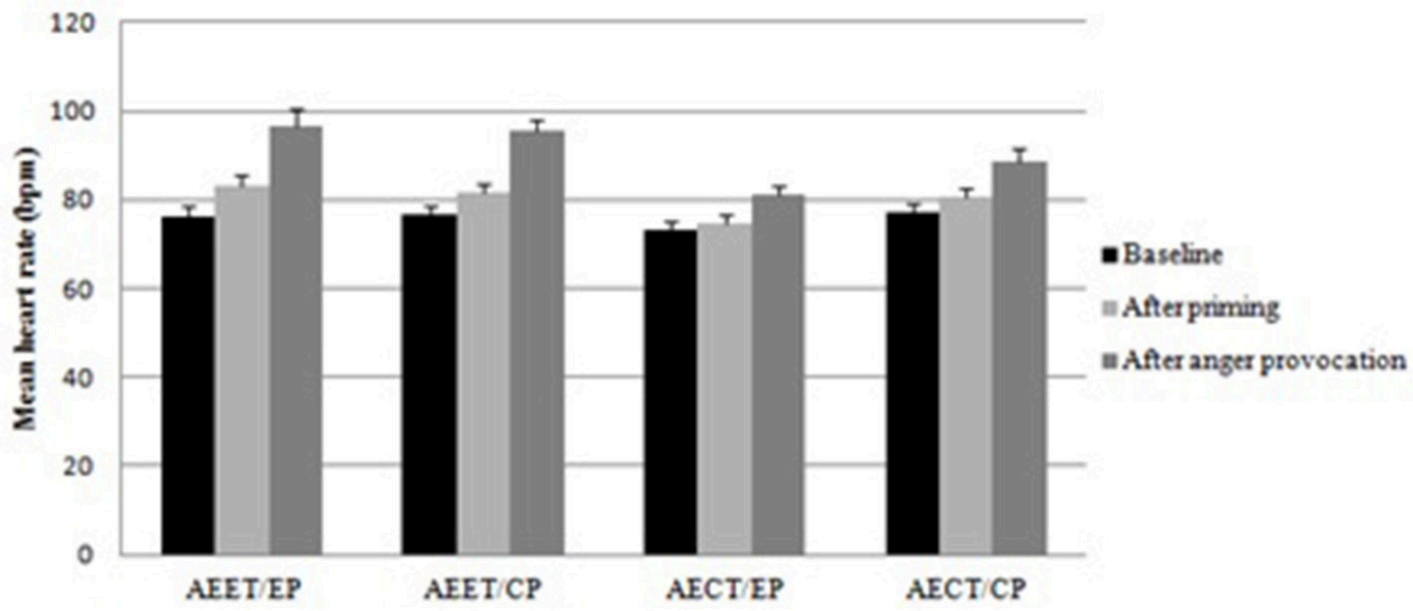
**Mean of heart rate for the four groups (AEET/EP, automatic emotion expression tendency/expression priming; AEET/CP, automatic emotion expression tendency/control priming; AECT/EP, automatic emotion control tendency/expression priming; AECT/CP, automatic emotion control tendency/control priming) across the three measurement points (baseline, after priming, after anger provocation; error bars represent standard errors of the mean)**.

Heart rate data are displayed in Figure 3. The analysis revealed a main effect of automatic emotion expression tendency for the heart rate after the anger provocation, *F*_(1, 94)_ = 13.815, *p* < 0.001, η_*p*_^2^ = 0.134. The participants with automatic expression tendencies had higher heart rates than participants with automatic control tendencies (*p* < 0.01). The same pattern was also found for the heart rate after priming, main effect of automatic emotion expression tendency, *F*_(1, 94)_ = 8.171, *p* < 0.01, η_*p*_^2^ = 0.084. Participants with automatic expression tendencies had higher heart rates than participants with automatic control tendencies after priming (*p* < 0.05). The means and SD of heart rate were presented in Table 1.

## Discussion

This study investigated whether individual difference in AER and an AER priming manipulation interact in their effects on emotion regulation during an anger provocation. We found that individuals with automatic emotion control tendencies primed to control emotion showed a lower SCL than individuals with automatic emotion control tendencies primed to express emotion only after anger provocation, not in the priming period. However, the SCL of individuals with automatic emotion expression tendencies showed no difference in the control priming or the expression priming condition. This pattern supports the hypothesis that individual differences in AER interact with the primed emotional regulation strategy. Heart rate of individuals with automatic emotion expression tendencies was higher than that of individuals with automatic emotion control tendencies in response to the anger provocation regardless of the emotion regulation priming condition. These results indicated that the SCL was sensitive to the interactive effects of individual differences in AER and AER priming. These results also provided an indication that heart rate might be responsible for individual differences in AER but not AER priming.

Emotion regulation priming differentially affected SCL for persons with automatic emotion control tendencies, but had a uniform effect for persons who displayed automatic emotional expression tendencies. This result revealed that the SCL of the group of automatic emotional control individuals would be changed by priming regulation goals. In the anger provocation, they increased SCL under the emotion expression priming and decreased SCL under the implicit goal of emotion control. The SCL of the group of automatic emotion expression individuals were very high either in emotion control priming or in emotion expressing priming. SCL, which reflects sympathetic nervous activity, is a reliable and sensitive index of emotional arousal and arousal changes (Boucsein, 1992; Lang, 1995; Khalfa et al., 2002; Zhang et al., 2011). A possible explanation would be that a conflict between primary emotion regulation tendency and primed regulation goal (such as in participants with automatic emotion control tendencies who are faced with expression priming, or participants with automatic emotion expression tendencies who are faced with control priming), may engender negative feelings (Gorges et al., 2014) which can increase SCL (Khalfa et al., 2002). Thus, the SCL increased significantly when participants with automatic emotion control (or expression) tendency were primed by the inconsistent emotion regulation goal. These results indicated that the effect of AER was modulated by the consistency of individuals' regulation tendency and the priming regulation goal. If the priming regulation goal was inconsistent with individuals' regulation tendency, AER would increase the sympathetic nervous reaction.

The heart rate results of the present study are consistent with prior findings of lower heart rate in participants with automatic emotion control tendencies relative to participants with automatic emotion expression tendencies during anger (Mauss et al., 2006). More importantly, we found that this difference in response to anger was not affected by the emotion regulation priming manipulation. Heart rate, which is an index of joint sympathetic and parasympathetic activation, would increase for anger induction and is sensitive to emotional valence (Campbellsills et al., 2006; Hofmann et al., 2009; Herrero et al., 2010). It has been documented that automatic processes decreased the need for elevated cardiac outputs to supply active neural structures with glucose (Kennedy and Scholey, 2000; Christou-Champi et al., 2015). Our result suggests that individuals with automatic emotion control recruit fewer resources during the provocation which negated the need for elevated cardiac output when anger was provoked. Alternatively, the findings may also provide an indication that the heart rate is less sensitive to the effects of emotion regulation related priming manipulations.

It is interesting to note that no significant effects of emotion priming condition or an interaction with individual difference in AER was found in heart rate, although the increase in self-reported anger was lower in the control priming condition. However, the current result is consistent with the findings of Mauss et al. (2007b) who reported a reduction in self-reported anger after emotion control priming in absence of a difference in cardiovascular responses. This pattern indicated that emotion regulation priming would reduce the anger feelings cost freely.

The interaction of priming and individual differences in the present study has implication for research using priming interventions to stimulate emotion regulation. Previous studies indicated that priming regulation goals could activate regulation tendencies and influence emotional responses unconsciously (Fitzimons and Bargh, 2004; Koole and Coenen, 2007; Mauss and Tamir, 2014; Kobylińska and Karwowska, 2015). Extending previous findings, the present study suggested that when the regulation goals were inconsistent with individuals' regulation tendency, participants would be emotional aroused. Thus, it is reasonable for studies on AER which use priming control for individual differences in AER. The present study also suggests that clinical practice or training of emotion regulation skills (for example, reappraisal training; Kobylińska and Karwowska, 2015), should consider individuals' automatic regulation tendency by assessing automatic emotion control and expression tendencies and designing interventions accordingly.

The current study has two limitations which can qualify the results. First, we could not examine whether the sentence unscrambling task indeed primed the different emotion regulation goals. The sentence unscrambling task was adapted from Mauss et al. (2007b) and we did not include a manipulation check as to whether this task primed participants' regulation goals as this might have interfered with the effects of priming during the subsequent anger provocation. Second, we only assessed the effects of individual differences in AER and emotion regulation priming in response to an anger provocation. The findings should be replicated in reference to other emotions, such as fear, happy, sadness, and disgust. Finally, to provide stronger implications for clinical practice, future studies should examine a range of regulation strategies and apply the techniques from cognitive neuroscience to examine the neural base of AER.

## Conclusion

In an anger provocation situation, individual differences in AER interacted with emotion regulation priming to affect SCL, whereas heart rate were not affected by emotion regulation priming. Specifically, SCL was lower in individuals with automatic emotion control tendencies in the control priming condition than in those with automatic emotion control tendencies in the expression priming condition. However, emotion regulation priming conditions did not affect the SCL of individuals with automatic emotion expression tendencies. HR was elevated during the anger provocation in participants with automatic emotion expression tendencies. The current study emphasizes the importance of individual differences in AER tendencies and suggests that they need to be considered in future studies of emotion regulation.

## Author contributions

JZ drafted the manuscript. OL and PH provided critical revisions. JZ, OL, and PH conceived and designed the experiments. JZ and PH performed the experiments. JZ, OL, and PH analyzed the data. JZ, OL, and PH provided the explanation of the data.

## Funding

The research was supported by a grant from the National Natural Science Foundation of China (31200845), the Fundamental Research Funds for the Central Universities and the Research Funds of Renmin University of China (15XNL029) granted to JZ, and partly supported by the Australian Research Council (DP150101540) to OL.

### Conflict of interest statement

The authors declare that the research was conducted in the absence of any commercial or financial relationships that could be construed as a potential conflict of interest.

## Appendix A

The regulation words applied in the Sentence Unscrambling Task

Control words: 冷静(calm)掩盖(cover)隐藏(hide)藏锁(seal)关闭(close)控制(control)压制(suppress)规划(plan)平静(quiet)

Expression words: 冲动(impulse)揭露(expose)表达(express)泄露(reveal)敞开(open)开放(unrestricted)放纵(indulge)起伏(undulate)喷出(blowout).

## Appendix B

The words used in the ER-IAT

Negative category: 消极(Negative)、 讨厌(Filth)、 肮脏(Rotten)、 阴暗(Gloom)、 糟糕(Bad)

Positive category: 积极(Pleasant)、 良好(Good)、 幸运(Lucky)、 阳光(Gold)、 光荣(Honor)

Emotion expression: 释放(Discharge)、 动情(Emotional)、 吐露(Reveal)、 表达(Expressive)、 流露(Disclose)

Emotion control: 控制(Controlled)、 压制(Suppress)、 包容(Contain)、 隐藏(Hide)、 冷静(Cool).
